# An acute acalculous cholecystitis in a returned travel couple

**DOI:** 10.1371/journal.pntd.0006177

**Published:** 2018-03-08

**Authors:** Daan A. R. Castelijn, G. H. Wattel-Louis

**Affiliations:** Department of Internal Medicine, Spaarne Gasthuis Medical Centre, Hoofddorp, the Netherlands; Yale University Yale School of Public Health, UNITED STATES

## Case description and question

**Fig 1:**
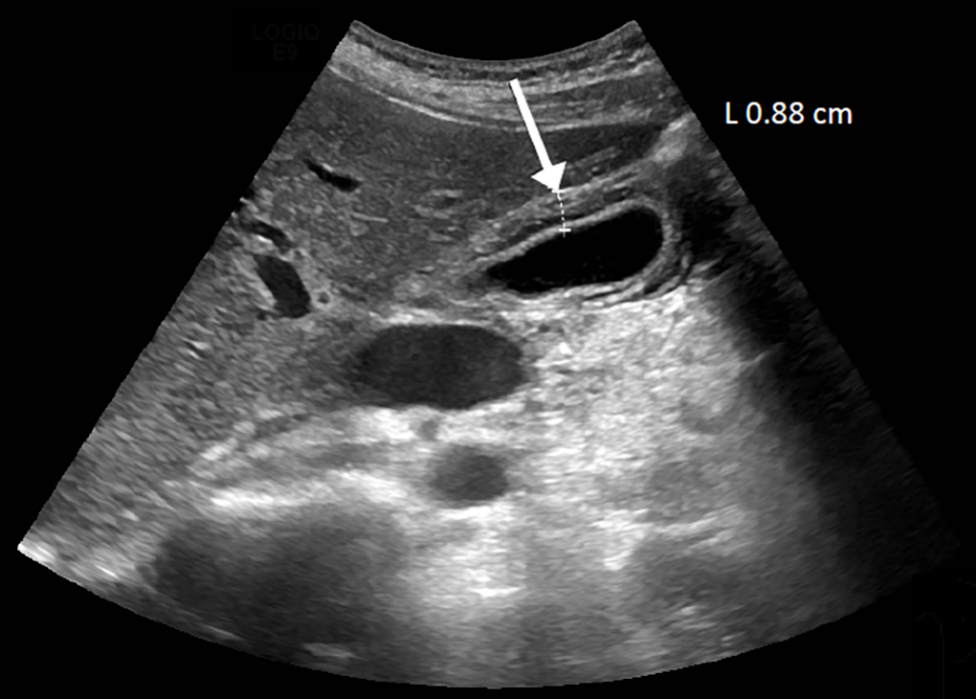
Abdominal ultrasound shows signs of an AAC with gall bladder wall thickening (white arrow). AAC, acute acalculous cholecystitis.

A previously healthy 35-year-old Swiss woman presented with fever, headache, and myalgia for three days. Symptoms began on the final day of travel in Colombia. Abdominal examination revealed a positive Murphy sign. Laboratory results at presentation are shown in Table 1. Abnormal findings included a thrombocytopenia (121,000/mm^3^), an elevated total bilirubin (48 μmol/liter), and high alkaline phosphatase (163 U/liter). Abdominal ultrasound showed signs of an acute acalculous cholecystitis (AAC) (Fig 1).

**Table 1:** Laboratory results at presentation.

**Laboratory value (unit)**	**Reference range**	**Female patient**	**Male patient**
Hemoglobin (mmol/liter)	Female: 7.5–10.0 Male: 8.5–11.0	7.8	8.1
Platelet count (per mm^3^)	150,000–400,000	121,000	121,000
White blood cell count (per mm^3^)	4,000–10,000	10,900	4,400
Creatinine (μmol/liter)	Female: 49–90 Male: 64–104	81	102
Urea nitrogen (mmol/liter)	2.5–6.4	5.1	5.5
Bilirubin (μmol/liter) Total Direct	3–20 0–4	48 36	34 24
Alkaline phosphatase (U/liter)	0–98	163	163
Gamma-glutamyltransferase (U/liter)	0–55	150	334
Alanine aminotransferase (U/liter)	0–34	97	214
Aspartate aminotransferase (U/liter)	0–31	79	174
Creatine kinase (U/liter)	0–171	33	28
C-reactive protein (mg/liter)	0–5	114	241

**Fig 2:**
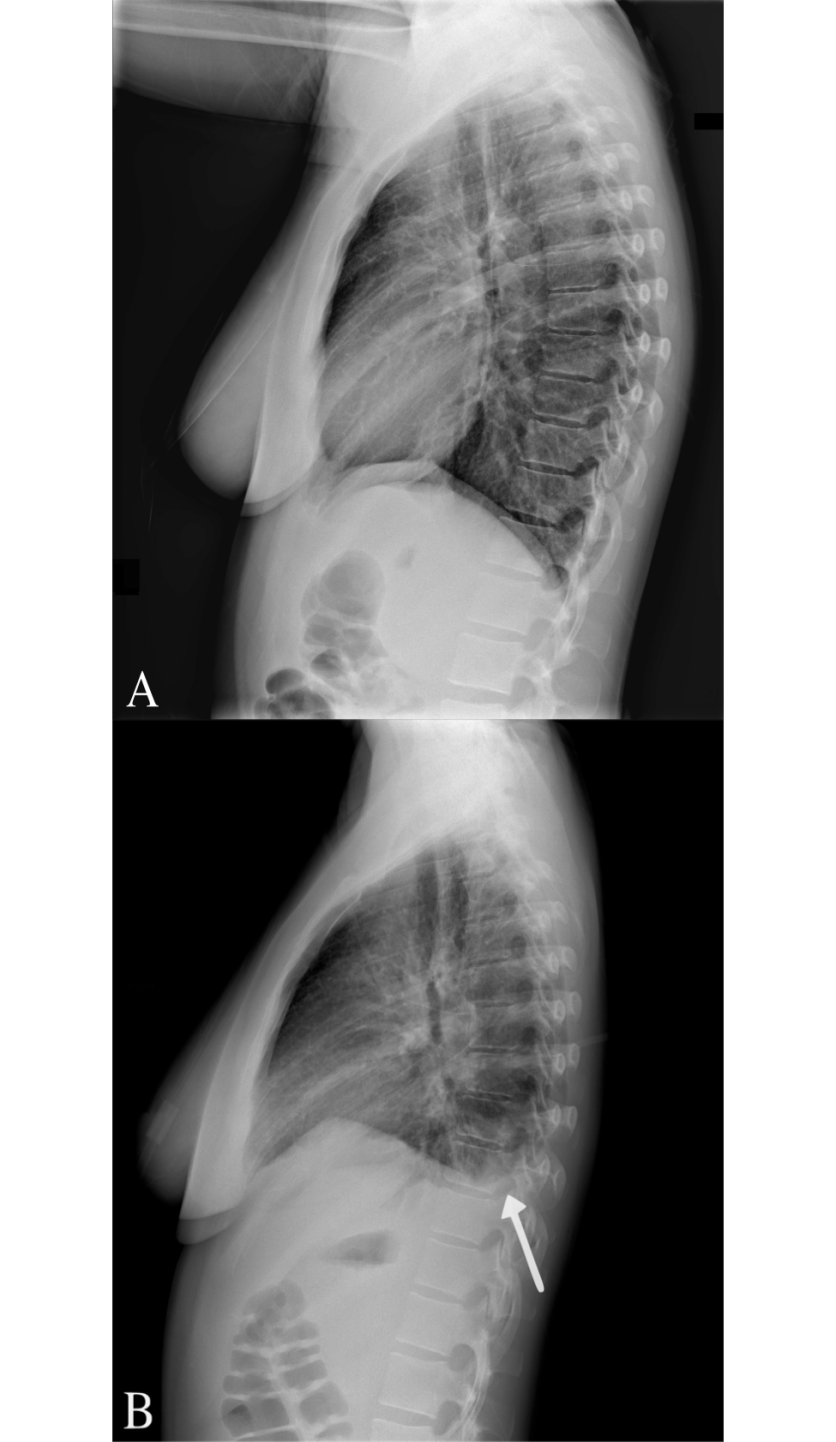
Radiography shows development of an infiltrate. (A) Lateral chest radiography at admission to the hospital shows no abnormalities. (B) Three days later, radiography reveals a left-sided infiltrate (white arrow).

At presentation, chest radiography showed no abnormalities (Fig 2A). On the third day of hospitalization, however, the patient developed a cough, dyspnea, and hypoxemia. A second chest radiography revealed a left-sided infiltrate (Fig 2B). Her traveling companion was admitted to the hospital with identical symptoms, including an AAC and development of pneumonia. What is your diagnosis?

## Answer and discussion

The diagnosis is leptospirosis. An infectious aetiology was suspected as both patients had an identical clinical presentation, including an AAC. An important diagnostic clue was that the couple had rafted in Colombia two weeks prior to the onset of symptoms. Rafting in fresh water is a well-known risk factor for leptospirosis [1]. Both patients had been rafting in the Fonce River in northeastern Colombia, where leptospirosis has been described [2]. The patient’s leptospirosis IgM antibody titer was dubiously reactive at 1:40 three days after the onset of symptoms. After treatment, the patient returned to Switzerland. Her companion’s serum was PCR positive for leptospirosis. Subsequent genotyping of the PCR product identified *Leptospira borgpetersenii* as the likely infecting species. The IgM titer was 1:40 at presentation, but at day 40, seroconversion had occurred, and the IgM titer was strongly positive at 1:640. The microscopic agglutination test (MAT) showed a high titer with serovar Mini from serogroup Mini, so the presumptive infecting serogroup is Mini. This specific serogroup has been shown to occur in Colombia [3]. Unfortunately, the culture proved negative, so the pathogenic serovar could not be determined. Serology for human immunodeficiency virus (HIV) type 1 and type 2 were negative. Both patients were treated with amoxicillin and clavulanate for 10 days, initially because of high fever and concern about abdominal sepsis. The symptoms gradually resolved during conservative treatment, and no oliguria or renal failure occurred. A cholecystectomy was not performed.

AAC accounts for approximately 5%–10% of all cases of acute cholecystitis and is potentially caused by infections. Bacterial pathogens associated with AAC are *Salmonella* spp., *Leptospira* spp., *Brucella* spp., *Rickettsia* spp., and *Coxiella burnetii*. Viral agents that have been described to cause AAC are hepatitis A virus, hepatitis B virus, cytomegalovirus, Epstein–Barr virus, and dengue virus [4]. Besides leptospirosis, a possible concomitant infection of these viruses and bacteria tested negative in our patients. The literature on AAC as the presenting symptom of leptospirosis is sparse, although some case reports have emphasized the relationship [5–7]. A proposed pathogenesis is an immunological response to the *Leptospira* infiltrating the gall bladder. A previous study performed histopathological examination of the gall bladder that revealed endothelial damage and submucosal oedema. Immunohistochemistry showed a *Leptospira* spirochete [8]. Too few cases have been reported to ascertain the most frequent *Leptospira* serovars associated with AAC. After review of the literature, one study concluded that in 6 of 14 reported cases, serovars belonging to *L*. *interrogans* were identified as the cause of AAC [9]. Furthermore, this study describes a case of AAC caused by *L*. *borgpetersenii*, similar to the causative species in our patients.

Both patients developed pneumonia exactly six days after the initial onset of symptoms. Pulmonary involvement in leptospirosis ranges from 20%–70% [10]. Pulmonary symptoms usually begin between the fourth and sixth day of disease [11]. Intra-alveolar hemorrhage and acute respiratory distress syndrome (ARDS) can develop as severe manifestations of pulmonary leptospirosis. However, in our patients, pulmonary symptoms remained mild and responded adequately to treatment.

This case is a reminder that leptospirosis is essential in the differential diagnosis of an AAC and that both biliary and pulmonary involvement are possible in the disease course.

## Ethics statement

Both patients gave consent to have their case details published.

**Key learning points**

- An acute acalculous cholecystitis is a potential manifestation of leptospirosis.
- Rafting is a well-known risk factor for leptospirosis.
- Both biliary and pulmonary involvement is possible in the disease course of leptospirosis.
